# Current landscape and challenges in anaerobic bloodstream infections: epidemiology, microbiology, and clinical management

**DOI:** 10.3389/fmicb.2026.1791932

**Published:** 2026-04-07

**Authors:** Ke Zhou, Huabin Wang, Yijun Zhu

**Affiliations:** Department of Clinical Laboratory, Affiliated Jinhua Hospital, Zhejiang University School of Medicine, Jinhua, Zhejiang, China

**Keywords:** anaerobic bloodstream infections, antimicrobial resistance, diagnostic challenges, epidemiology, therapeutic strategies

## Abstract

Anaerobic bloodstream infections (AnBSI) are insidious and potentially life-threatening conditions. Despite advances in diagnostics and therapy, the clinical management of AnBSI continues to face significant challenges. This review synthesizes current evidence on the epidemiology, evolving microbiology, antimicrobial resistance trends, clinical presentations, and management strategies for AnBSI. Persistent obstacles include pronounced global disparities in epidemiological data, a critical lack of rapid antimicrobial susceptibility testing methods, a scarcity of high-quality evidence to inform treatment, and ongoing under-recognition in clinical practice. To bridge these gaps, future initiatives must prioritize the development of stratified, practical guidelines that promote molecular diagnostics in high-resource settings while ensuring the routine implementation and standardization of anaerobic cultures in resource-limited settings. Concurrently, the immediate implementation of a bundled anaerobic blood culture collection strategy for high-risk populations stands as the most cost-effective intervention currently available.

## 1 Introduction

The effective management of bloodstream infections (BSI) is a critical determinant of patient survival, as delays in pathogen identification and antimicrobial susceptibility testing are directly associated with worsened outcomes (Torvikoski et al., 2025). Anaerobic bloodstream infections (AnBSI) represent a distinct and particularly challenging subset of BSIs. This distinctiveness arises from the unique nature of the pathogens (obligate anaerobes with fastidious growth requirements), inherent diagnostic complexities (necessitating specialized culture conditions and frequently missed by routine aerobic methods), and the specific demands of clinical management, where empiric therapy must reliably cover anaerobic organisms (Diallo et al., 2025; Di Bella et al., 2022; Nagaoka et al., 2024; Ransom and Burnham, 2022; Vergara et al., 2024).

Clinically, AnBSI often presents with insidious and non-specific symptoms, typically originating from breaches in the gastrointestinal or female genital tract barriers in hosts with significant comorbidities (Yang et al., 2024). Historical under-recognition and diagnostic delays are primarily attributable to two interrelated challenges: the fastidious nature of anaerobic pathogens and the inconsistent application of optimal diagnostic methods, such as dedicated anaerobic blood culture bottles (Temkin et al., 2022; Vergara et al., 2024). Consequently, patients face an elevated risk of severe complications and mortality, highlighting the imperative for heightened clinical suspicion and optimized diagnostic pathways (Kierzkowska et al., 2025; Murai et al., 2025; Reissier et al., 2023).

Furthermore, the therapeutic landscape is evolving due to the concerning emergence of antimicrobial resistance among anaerobes, which threatens the efficacy of established cornerstone therapies, as evidenced by contemporary surveillance data (Boattini M. et al., 2025b; Buhl et al., 2024; Kovács et al., 2022; Reissier et al., 2023). This trend underscores the urgent need for a paradigm shift toward more precise, evidence-based, and rapid diagnostic-therapeutic strategies.

This review aims to synthesize current evidence on the epidemiology, evolving microbiology, clinical presentation, and therapeutic management of AnBSI. By examining the extant knowledge base, identifying persistent evidence gaps, and proposing a strategic framework for future research and clinical optimization, this article charts a course toward improving outcomes in the management of these complex infections. The integration of innovative concepts such as “microbiologistics”—which seeks to holistically optimize the diagnostic workflow from sample collection to result reporting—is posited as crucial for maximizing the real-world impact of advancing technologies on patient care (Minami et al., 2022; Valencia-Shelton et al., 2024).

## 2 Epidemiological profile: heterogeneity and high-risk associations

The epidemiology of anaerobic bloodstream infections (AnBSI) exhibits marked geographical and demographic heterogeneity, underscoring the necessity for context-specific clinical awareness. Large-scale surveillance initiatives, such as the comprehensive Italian national study (ITANAEROBY), have been instrumental in delineating local pathogen distributions and quantifying disease burden (Di Bella et al., 2022). A critical epidemiological insight is the well-established association between AnBSI and occult colorectal malignancy, consistently demonstrated across multiple cohort studies (Justesen et al., 2022, 2024). This relationship elevates AnBSI from a mere infectious complication to a potential diagnostic indicator of underlying gastrointestinal pathology, necessitating a comprehensive diagnostic work-up in suitable patient populations (Justesen et al., 2024).

## 3 Pathogen spectrum and escalating antimicrobial resistance

Contemporary surveillance data from several regions, including comprehensive national studies in Denmark and broader European multicenter analyses, reveal a concerning trend of increasing antimicrobial resistance among anaerobic pathogens (Buhl et al., 2024; Florisson et al., 2025). A critical caveat is that current high-quality epidemiological and resistance surveillance data demonstrate a profound global disparity, being overwhelmingly concentrated in North America and Europe (Buhl et al., 2024; Florisson et al., 2025; Reissier et al., 2023). Significant reporting gaps persist from Asia, Africa, and South America. This inequity not only limits the generalizability of international management guidelines but also obscures the true global burden and resistance landscape of AnBSI. Resistance to key agents such as metronidazole and carbapenems, while still relatively uncommon, is being reported with growing frequency, notably within the *Bacteroides fragilis* group (Reissier et al., 2023). This trend underscores the imperative for robust, ongoing local surveillance and prompt antimicrobial susceptibility testing to inform effective therapeutic strategies (Murai et al., 2025).

## 4 Clinical characteristics: from subtle signs to distinct syndromes

The clinical presentation of anaerobic bloodstream infections (AnBSI) is highly variable, spanning from an insidious, non-specific onset to fulminant sepsis. A recent large multicenter retrospective cohort study specifically delineated the clinical phenotype of anaerobic bacteremia in the absence of detectable abscess lesions, underscoring the unique management challenges in such cases (Nagaoka et al., 2024). Notably, polymicrobial infections are common and have been independently associated with more severe clinical courses and higher mortality (Acker et al., 2022; Hosoda et al., 2025). These polymicrobial AnBSI most frequently involve a combination of anaerobic Gram-negative bacilli and facultative anaerobes. The pathogenic synergy between these organisms is considered a core driver of the poor prognosis, which in turn necessitates broader-spectrum coverage in empiric antimicrobial therapy (Acker et al., 2022; Hosoda et al., 2025). While specific syndromes such as Lemierre's syndrome (caused by *Fusobacterium necrophorum*) present with characteristic clinical features, such classic and distinct presentations are the exception rather than the rule (Foo et al., 2021; Hemani et al., 2022).

## 5 Diagnostic advances: accelerating time to knowledge

Optimizing the diagnostic pathway is paramount to improving outcomes in anaerobic bloodstream infections (AnBSI). The foundational step is ensuring proper specimen collection; robust evidence supports the routine use of dedicated anaerobic blood culture bottles, which significantly enhances pathogen recovery and reduces the time to detection, a practice validated across both adult and pediatric populations (Gottschalk et al., 2024; Kato et al., 2023; Ransom and Burnham, 2022).

Matrix-assisted laser desorption/ionization time-of-flight mass spectrometry (MALDI-TOF MS) has revolutionized the identification of anaerobic bacteria from positive blood cultures, dramatically shortening turnaround times compared to conventional methods (D'Inzeo et al., 2021; Watanabe et al., 2021). Direct identification from blood culture bottles using optimized MALDI-TOF MS protocols shows high concordance with traditional biochemical techniques, offering a rapid and reliable alternative (Schmitt et al., 2013). This is particularly advantageous for identifying fastidious pathogens such as *Fusobacterium and Clostridium* species (Watanabe et al., 2021).

Looking forward, clinical metagenomic next-generation sequencing (mNGS) holds significant promise. By enabling culture-independent, direct detection of pathogens and resistance markers from blood, mNGS can deliver comprehensive diagnostic information far more rapidly than conventional workflows (Grumaz et al., 2016). Its unique value lies in diagnosing culture-negative AnBSI, substantially increasing diagnostic yield and overcoming the limitations of culture in detecting polymicrobial and resistant infections (Shi et al., 2026).

Other emerging technologies, such as pyrolysis-gas chromatography-ion mobility spectrometry (Py-GC-IMS) and ultraviolet photoionization time-of-flight mass spectrometry, are under investigation for potential rapid, direct specimen identification (Kobelt et al., 2025; Li et al., 2025).

The expanding array of diagnostic tools—each with a distinct profile of speed, accuracy, cost, and complexity—necessitates a structured, strategic approach to their integration into clinical practice (see Table 1).

**Table 1:** Comparison of performance characteristics of identification methods for anaerobic bloodstream infections.

Method	Time to result (after sample receipt)	Key advantages	Key disadvantages	Application	References
Conventional culture	2–7+ days	Gold standard, provides isolate for full AST, low cost per test	Very slow, sensitivity affected by prior antibiotics	Routine diagnostics where AST is mandatory; resource-limited settings	(Caliendo et al., 2013; Lamy et al., 2016; Ombelet et al., 2019)
MALDI-TOF MS	1–2 days (after growth)	Rapid, accurate identification from colonies; cost-effective per ID	Does not speed up initial detection; requires growth	Routine, rapid identification of grown isolates	(D'Inzeo et al., 2021; Schmitt et al., 2013; Watanabe et al., 2021)
Broad-range PCR/sequencing	6–48 h	Identifies uncultivable organisms; high sensitivity/specificity	No AST; high cost and expertise; risk of contamination	Culture-negative cases or definitive ID of unusual isolates	(Behera et al., 2021; Cherkaoui et al., 2009; Lieu et al., 2024)
Multiplex PCR	1–3 h	Very fast detection & ID of common pathogens + resistance markers	Limited target menu; no isolate; high reagent cost	Rapid simultaneous detection of multiple pathogens	(Kim et al., 2024; Serapide et al., 2025; Yilmaz et al., 2025)
mNGS	2–5 days	Unbiased, detects all potential pathogens in a single test	Very high cost, complex analysis, unclear routine utility	Research or complex, culture-negative cases	(Blauwkamp et al., 2019; Grumaz et al., 2016; Shi et al., 2026)
Emerging tech (e.g., Py-GC-IMS, UV-PI-TOFMS)	1–2 days (after growth)/Potential for minutes	Ultra-fast, label-free analysis; potential for direct-from-broth analysis	Early R&D stage; lacks validated clinical databases; not standardized	Proof-of-concept research; potential for future point-of-care or rapid screening	(Kobelt et al., 2025; Li et al., 2025)

**Figure 1:**
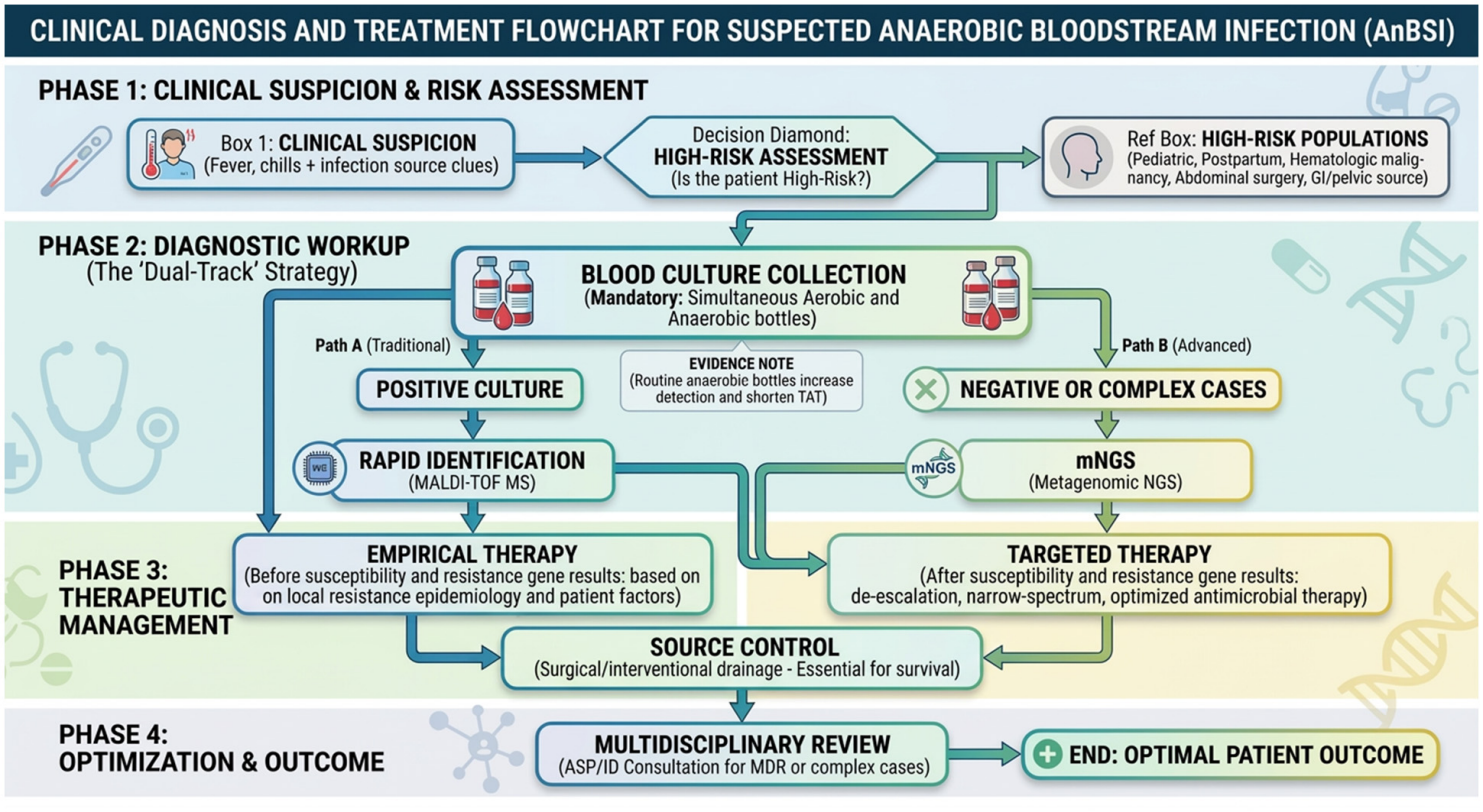
Clinical Diagnosis and Treatment Flowchart for Suspected Anaerobic Bloodstream Infection (AnBSI). AnBSI, Anaerobic Bloodstream Infection; GI, Gastrointestinal; MALDI-TOF MS, Matrix-Assisted Laser Desorption/Ionization Time-of-Flight Mass Spectrometry; mNGS, Metagenomic Next-Generation Sequencing; TAT, Turnaround Time; MDR, Multidrug Resistant; ASP, Antimicrobial Stewardship Program; ID, Infectious Diseases.

To translate these technological advancements into actionable clinical practice and address the persistent challenges in AnBSI diagnosis and management, we propose a stepwise clinical management framework (Figure 1). This framework is conceptually built upon three interdependent pillars: evidence-based clinical decision-making, stratified risk management, and integrated diagnostic-therapeutic collaboration.

The structure and logic of each step within the framework are derived from a synthesis of: (i) key evidence from high-quality cohort studies identifying actionable risk factors and outcomes (Di Bella et al., 2022; Ransom and Burnham, 2022; Torvikoski et al., 2025); (ii) established expert recommendations for optimizing diagnostic yield and therapeutic efficacy (Temkin et al., 2022; Valencia-Shelton et al., 2024); and (iii) comprehensive antimicrobial resistance surveillance data guiding empirical choices (Di Bella et al., 2022; Reissier et al., 2023). Consequently, every step is designed to address a specific, well-documented clinical gap in AnBSI care, such as under-recognition, diagnostic delay, and uninformed empirical therapy.

The resulting flowchart formalizes a three-phase management pathway, each underpinned by specific clinical reasoning and evidence:

1. Clinical suspicion and risk assessment
2. This initial phase is grounded in the epidemiological principle of targeting high-yield populations to improve early detection. It begins with the recognition of non-specific signs (e.g., fever, chills) but critically incorporates active risk stratification. The listed high-risk criteria (e.g., pediatric/neonatal status, hematologic malignancy, and recent abdominal surgery) are not arbitrary; they are consistently identified in clinical studies as carrying a significantly higher pre-test probability for AnBSI (Di Bella et al., 2022; Nagaoka et al., 2024; Yang et al., 2024). Triggering a diagnostic workup based on this stratification is a consensus strategy to mitigate the common pitfall of diagnostic delay (Ransom and Burnham, 2022; Temkin et al., 2022).
3. Diagnostic workup
4. The recommended diagnostic sequence follows a hierarchical, evidence-based logic to maximize pathogen recovery and identification efficiency. The mandate for paired aerobic/anaerobic blood cultures is supported by robust evidence (Class I) demonstrating its superiority over aerobic-only cultures in recovering anaerobic pathogens, forming the non-negotiable foundation of diagnosis (Murai et al., 2025; Ransom and Burnham, 2022). For positive cultures, the immediate use of MALDI-TOF MS represents the consensus standard for rapid, cost-effective identification (D'Inzeo et al., 2021; Watanabe et al., 2021). The framework then logically incorporates mNGS as a targeted, advanced option for culture-negative or complex cases, reflecting a solution for a defined diagnostic dilemma where conventional methods fail (Blauwkamp et al., 2019; Shi et al., 2026). This creates a “tiered diagnostic strategy” that balances speed, cost, and diagnostic breadth (Diallo et al., 2025; Shi et al., 2026).
5. Therapeutic management
6. This phase operationalizes the principle of dynamic, information-driven therapy. It explicitly differentiates between empirical and targeted therapy stages, a distinction based on the fundamental clinical need to balance broad initial coverage with subsequent precision. Empirical therapy recommendations are directly tied to likely pathogen profiles based on infection source and local resistance patterns (Murai et al., 2025; Reissier et al., 2023). The transition to targeted therapy, guided by susceptibility data, is emphasized as a cornerstone of antimicrobial stewardship and is key to improving outcomes and curbing resistance (Kierzkowska et al., 2025). Furthermore, the framework elevates source control (a Class II recommendation) and multidisciplinary collaboration (via ASPs and ID consultation) from supportive measures to essential, integrated components of care, based on their documented impact on mortality, especially in focal infections like those of abdominal origin (Khankan et al., 2025; Kierzkowska et al., 2025; Valencia-Shelton et al., 2024).

## 6 Therapeutic strategies: navigating uncertainty

The management of anaerobic bloodstream infections (AnBSI) is characterized by a significant evidence gap, with current strategies predominantly informed by observational studies, expert consensus, and local resistance epidemiology rather than high-level clinical trial data (Murai et al., 2025; Reissier et al., 2023; Wareham et al., 2005). This deficit creates substantial uncertainty in several pivotal areas of clinical decision-making. For crucial questions regarding the optimal choice of empirical agent (e.g., carbapenems vs. beta-lactam/beta-lactamase inhibitor combinations), the definitive role and composition of combination therapy, the optimal duration of treatment, and the specific impact of source control, clinicians must often rely on low-grade evidence and extrapolation (Murai et al., 2025; Wareham et al., 2005).

For first-line empirical therapy, carbapenems (e.g., meropenem, imipenem) and beta-lactam/beta-lactamase inhibitor (BLBLI) combinations (e.g., piperacillin-tazobactam, amoxicillin-clavulanate) constitute the mainstay. Available comparative data suggest comparable clinical efficacy for metronidazole-susceptible AnBSI (Murai et al., 2025; Reissier et al., 2023). However, this apparent equivalence requires nuanced interpretation. Carbapenems are generally preferred in settings with high rates of beta-lactamase-producing *Bacteroides spp*. or for infections involving metronidazole-resistant *B. fragilis* group isolates, against which they demonstrate superior *in vitro* activity and clinical outcomes (Murai et al., 2025; Reissier et al., 2023). The choice between these classes must be individualized, integrating the likely infection source, local resistance patterns, and patient-specific factors (e.g., renal function, allergy history; Khankan et al., 2025; Reissier et al., 2023). This source-driven approach is critical: AnBSI of gastrointestinal origin, predominantly caused by the *Bacteroides fragilis* group and *Clostridium* species, necessitates reliable coverage of anaerobic Gram-negative bacilli. In contrast, AnBSI stemming from the female genital tract, frequently involving *Peptostreptococcus* and *Prevotella* species, may be adequately addressed by certain BLBLI combinations, which also provide coverage for common co-pathogens (Reissier et al., 2023; Yang et al., 2024).

The role of combination therapy remains controversial and is typically reserved for severe presentations such as septic shock, or for polymicrobial infections where broader coverage is desired. The empirical addition of metronidazole to a carbapenem is a common practice, intended to ensure complete anaerobic coverage and potentially provide synergy. However, robust evidence from randomized controlled trials (RCTs) confirming a mortality benefit for combination therapy over appropriate monotherapy in AnBSI is conspicuously lacking (Kierzkowska et al., 2025; Wareham et al., 2005). Its use thus represents a risk-averse strategy balanced against the principles of antimicrobial stewardship.

Determining the optimal treatment duration is primarily guided by the infection source and the patient's clinical and microbiological response. For uncomplicated AnBSI with prompt defervescence and no persistent focus, a course of 7–10 days is often sufficient (Kierzkowska et al., 2025; Murai et al., 2025). Conversely, for infections secondary to established intra-abdominal or pelvic abscesses, or in cases of persistent bacteremia, extended therapy of 14–21 days—or until adequate source control is achieved—is commonly required (Kierzkowska et al., 2025; Murai et al., 2025). Pragmatic dosing optimizations have been established; for example, a multicenter study validated that twice-daily intravenous metronidazole (15 mg/kg q12h) provides equivalent efficacy and safety to traditional thrice-daily dosing (7.5 mg/kg q8h), simplifying administration (Shah et al., 2021).

Source control is a non-negotiable cornerstone of management whenever anatomically feasible. Interventions such as surgical drainage, debridement, or removal of infected devices are paramount. In anaerobic bacteremia, particularly with *B. fragilis* group, failure to achieve timely source control has been independently associated with significantly increased mortality, underscoring its critical role beyond antimicrobial therapy alone (Kierzkowska et al., 2025).

Finally, antimicrobial stewardship programs (ASPs) are integral to optimizing AnBSI management. The integration of rapid diagnostic technologies, such as MALDI-TOF MS for immediate pathogen identification or metagenomic next-generation sequencing (mNGS) for culture-negative cases, can catalyze earlier de-escalation from broad empirical regimens. This has been shown to shorten the duration of unnecessary broad-spectrum therapy, reduce carbapenem exposure, and mitigate the risk of collateral resistance, thereby aligning treatment efficacy with stewardship imperatives (Khankan et al., 2025).

## 7 Persistent challenges and critical limitations

Despite significant diagnostic and therapeutic advances, the management of anaerobic bloodstream infections (AnBSI) continues to face substantial obstacles. These include the pronounced global disparities in epidemiological and antimicrobial resistance data previously highlighted (Buhl et al., 2024; Di Bella et al., 2022; Florisson et al., 2025), a critical lack of rapid antimicrobial susceptibility testing (AST) methods specifically developed for anaerobic bacteria (Li et al., 2025; Wareham et al., 2005), and a persistent scarcity of high-quality prospective clinical trials to guide therapeutic decisions (Kierzkowska et al., 2025; Murai et al., 2025; Wareham et al., 2005).

A fundamental barrier remains the under-recognition of AnBSI in clinical practice, frequently attributable to the omission of dedicated anaerobic blood cultures (Diallo et al., 2025; Ransom and Burnham, 2022; Temkin et al., 2022). This issue is exacerbated in low-resource settings, where the absence of specialized anaerobic culture bottles and a shortage of trained laboratory personnel contribute to high rates of misdiagnosis (Diallo et al., 2025). This diagnostic deficit starkly contrasts with the systematic surveillance and robust epidemiological infrastructure typical of high-income regions (Di Bella et al., 2022), thereby widening global inequities in AnBSI outcomes.

Furthermore, the challenge is dynamic. Even in well-resourced settings, the emerging trend of increasing antimicrobial resistance among anaerobes requires close monitoring (Florisson et al., 2025)—a concern that resource-limited health systems are even less equipped to manage, perpetuating the cycle of disparity.

## 8 Future directions and strategic recommendations

To address the aforementioned challenges, a multifaceted and strategic approach is imperative:

1. Establish a tiered and actionable management framework: the immediate priority is to implement a “bundled anaerobic blood culture collection” protocol for high-risk populations, representing the most cost-effective intervention currently available (Merien et al., 2023; Diallo et al., 2025; Minami et al., 2022; Torvikoski et al., 2025). For the medium to long term, it is essential to develop stratified, practical guidelines. These guidelines should promote the adoption of molecular diagnostic technologies in high-resource settings while ensuring the routine implementation and standardization of anaerobic blood cultures in resource-limited settings (Boattini et al., 2025a; Di Bella et al., 2022). This framework should be tailored based on regional resistance patterns and resource availability, adopting a “molecular-first” strategy where possible and prioritizing culture standardization where resources are constrained (Boattini et al., 2025a; Reissier et al., 2023).
2. Integrate innovative diagnostics and strengthen global surveillance: the diagnostic pathway should be optimized through an integrated “microbiologistics” model, actively incorporating next-generation tools like mNGS (Grumaz et al., 2016; Valencia-Shelton et al., 2024). Concurrently, efforts must be directed toward establishing a tiered global surveillance consortium. This network would integrate advanced centers (for deep sequencing and mechanistic research) with widely distributed sentinel sites (for core pathogen identification and resistance monitoring), prioritizing the sharing of critical resistance data and alerts concerning high-risk pathogens (Boattini et al., 2025a; Di Bella et al., 2022; Florisson et al., 2025). To enhance the global relevance of guidelines, this consortium should prioritize extending epidemiological surveillance to currently underrepresented regions (e.g., Asia, Africa, and South America) and establish unified AnBSI surveillance standards.
3. Launch targeted, prospective clinical trials: there is an urgent need for well-powered randomized controlled trials (RCTs) to address pivotal questions regarding comparative antibiotic efficacy, the utility of synergistic combinations, and the adequacy of shorter treatment courses in well-defined patient populations (Kierzkowska et al., 2025; Murai et al., 2025; Wareham et al., 2005). These trials should be stratified by infection source, pathogen, and disease severity to generate high-level evidence. International multi-center collaboration is crucial to overcome the sample size limitations inherent in single-center studies (Kierzkowska et al., 2025; Murai et al., 2025).
4. Deepen mechanistic and translational research: a deeper understanding of specific virulence factors (e.g., *Bacteroides fragilis* toxin) and the pathophysiology of complications is needed to unlock novel preventive strategies and targeted therapeutics (Choi et al., 2016; Grumaz et al., 2016; Reissier et al., 2023). For instance, identifying key virulence factors provides potential drug targets (Choi et al., 2016), and the application o technologies like CRISPR-Cas9 in anaerobic pathogen research may accelerate the development of targeted therapies (Grumaz et al., 2016).
5. Drive practice standardization through education and stewardship: sustained clinician education, the development of clear diagnostic and treatment algorithms, and the active engagement of Antimicrobial Stewardship Programs (ASPs) are necessary to ensure adherence to best practices (Khankan et al., 2025; Minami et al., 2022; Valencia-Shelton et al., 2024). Education should focus on improving recognition of high-risk populations and standardizing anaerobic culture collection. ASPs should develop AnBSI-specific protocols to guide rational antibiotic use, leveraging rapid diagnostic technologies to facilitate early de-escalation (Khankan et al., 2025; Minami et al., 2022).

In summary, anaerobic bloodstream infections represent a multifaceted and continually evolving challenge that cannot be resolved with simple, one-dimensional approaches. Advancing beyond the current limitations necessitates a deliberate and coordinated paradigm shift—from fragmented, observational practices to integrated, systematic action. This requires the synergistic convergence of enhanced global surveillance, innovative diagnostics, rigorous clinical research, and a steadfast commitment to standardizing care across all settings (Hosoda et al., 2025; Murai et al., 2025; Reissier et al., 2023).

Future efforts must be strategically phased with clear priorities: the immediate implementation of bundled anaerobic blood culture protocols; the medium-term development of resource-stratified guidelines and integration of advanced diagnostic technologies; and the long-term pursuit of high-level evidence through global collaborative research (Boattini et al., 2025a; Minami et al., 2022; Torvikoski et al., 2025; Wareham et al., 2005). Ultimately, only through such a sustained, coordinated, and multidisciplinary global effort can we meaningfully improve clinical outcomes for patients confronting these formidable infections (Grumaz et al., 2016; Reissier et al., 2023; Valencia-Shelton et al., 2024).
